# Effect of Axial Groove and Resin Luting Cements on the Retention of Complete Cast Metal Crowns

**Published:** 2009

**Authors:** K. Rajkumar, Aruna M. Bhat, Prasad D Krishna, Chetan Hegde, Manoj Shetty, N. Sridhar Shetty

**Affiliations:** * Assistant Professor, Al Ameen Dental College, Bijapur, Karnataka, India; ** Associate Professor, Department of Prosthodontics, A.B. Shetty Memorial Institute of Dental Sciences, Deralakatte-575018, Mangalore, Karnataka, India; *** Professor, Department of Prosthodontics, A.B. Shetty Memorial Institute of Dental Sciences, Deralakatte-575018, Mangalore, Karnataka, India; **** Professor, Director, CADDS, A.B. Shetty Memorial Institute of Dental Sciences, Deralakatte-575018, Mangalore, Karnataka, India

**Keywords:** Cementation, crowns, retention, resin cements, tensile strength

## Abstract

**Background:**

The design of the tooth preparation and the cementing medium are important consid-erations in the retention of crowns and fixed partial dentures. The purpose of this invitro study was to determine the effect of axial groove on the retention of complete cast metal crowns using two resin luting cements.

**Methods:**

Forty freshly extracted intact human molar teeth were prepared in their long axis to receive complete cast metal crowns. The specimens were randomly divided into two groups (one control and one study group). An axial groove of uniform size and shape was made on the prepared teeth under the study group. Axial surface area of prepared teeth specimens was measured. Complete cast metal crowns were fabricated for each specimen. Specimens of each group were divided into subgroups of 10 samples and were cemented with two resin luting cements, RelyX Unicem® and Calibra®, re-spectively. The cemented crowns were loaded in tension using a Universal Instron testing machine. The maximal tensile strength was recorded. Data were compared using the Mann-Whitney U test (α=0.05).

**Results:**

No significant differences in the tensile stress values were noted between the control (mean: 5.76±0.392 MPa) and study (mean: 5.93±0.751 MPa) groups cemented with RelyX Unicem. No significant differences in the tensile stress values were noted between the control (mean: 4.92±0.641 MPa) and study (mean: 5.15 ±0.478 MPa) groups cemented with Calibra. However, significant dif-ference in the tensile stress values was found between the two resin cements in the control and study groups.

**Conclusion:**

Axial groove placed in tooth preparations for resin bonded complete cast metal crowns had no statistically significant effect on retention. The use of (RelyX Unicem®) yielded greater retention values when compared to Calibra®.

## Introduction

The retentive strength of cemented cast metal crowns is important for their clinical success and depends on the operator’s tooth preparation^1^,^2^ and the cement used.^2^ The retention of extra-coronal restorations has been extensively discussed in dental literature.^3^,^4^ Dislodged cast restoration is a frequently encountered problem in clinical practice. Loss of crown retention was found to be one of the major causes of failure of traditional crowns and fixed partial dentures.^5^ Crown displacement often occurs because the features of the tooth preparation do not counteract the forces directed against the restorations.^6^ Therefore, the design of the tooth preparation is an important consideration in the retention of crowns and fixed partial dentures. It has been proved that retention is improved by geometrically limiting the number of paths along which a restoration can be removed from the tooth preparation. Maximum retention is achieved when there is only one path. A full veneer preparation with long, parallel axial walls and grooves would produce such retention.^7^ However, the influence of grooves of the prepared teeth on the retention of crowns has been discussed in the dental literature with varied results.^6^,^8^–^11^ Dental luting cements are the adhesive mediums between indirect restorations and prepared tooth surfaces. An ideal luting agent should provide a durable bond between dissimilar materials, posses sufficient compressive, tensile strength, and have sufficient fracture toughness to prevent dislodgement as a result of interfacial and cohesive failures.^12^

There are a wide variety of luting agents available with varied physical and chemical properties. Previous studies^13^,^14^ related to crown retention and type of luting agent have reported that resin luting cements provide greater retention than other contemporary dental luting agents. But, the results have been inconsistent.^15^,^16^

The strength of cement is a function of the bond and the mechanical properties of cements. The excellent bonding of adhesive resin luting cements to base metal alloys has been reported by Ergin^17^ and Browning.^13^ But, significant differences in the mechanical properties of the tested materials have been reported.^18^–^20^ The properties of resin cements are influenced by the nature of matrix, type of filler, filler volume, filler-matrix interfacial bond, filler load and polymerization mode.^21^,^22^ Adequate polymerization of the resin-based cement is an important prerequisite for the stability and biocompatibility of the restoration.^22^ Adhesive resin composite luting systems are furthermore recommended for the cementation of all-ceramic systems,^23^ but not metal-based fixed partial dentures, because of the possible risk of inadequate polymerization. Some modern resin composite luting cements can be cured by means of autopolymerization (self-curing) or by dual curing. However, there are not enough documented reports in the dental literature regarding the retention provided by these recently developed resin luting cements on the retention of cast metal restorations. The purpose of this study was to determine the effect of axial groove on the retention of complete cast metal crowns using two resin luting cements and to compare the retentive strengths of the conventional multistep dual cure resin cement with that of the self-adhesive universal resin luting cement.

## Materials and Methods

### 

#### Preparation of specimens

Forty freshly extracted intact human molar teeth of approximately same crown size were collected. Teeth were cleaned of surface debris, disinfected with 0.5% sodium hypochlorite and then, they were stored in distilled water at room temperature. The roots of all teeth were notched with a carborundum disk to resist dislodgement from the self-cured acrylic resin block when the specimens were tested in tension. The cementoenamel junction of each tooth was outlined using permanent marker. In order to align the tooth vertically, a Pindex system (Whaledent Pindex® System Mark II, Coltene/Whaltane Inc. USA) was used. The central beam light of the Pindex system was directed to locate the point of furcation of the roots of each tooth and this point was marked with permanent marker. Using Ney dental surveyor (Dentsply, USA), every tooth specimen was inversely aligned attaching the analyzing rod to the furcation point. The crown of each tooth was then embedded in Plaster of Paris up to 2 mm from the cementoenamel junction using a metal fixture. After setting of the plaster, another screw-retained metal fixture was placed over the first metal fixture and the roots of specimen teeth were then incorporated into self-cured acrylic resin aligning each tooth vertically parallel to its long axis. The occlusal surfaces of the teeth were flattened to expose the dentin using wheel diamond bur (No. 068-042. Sharpcut, Dentsply Asia). The axial reduction was carried out by using milling machine (Kavo-Elektrotechnisches Werk GmbH, type-990, West Germany) to standardize the degree of taper and finish line. A taper of approximately 6°C was produced in each axial wall with a round end tapered diamond bur (No. 111-014, Sharpcut, Dentsply Asia). The preparation margins were completed to a minimal (1 mm) wide circular chamfer finish line, 1-mm coronal to the CEJ. The bur was changed after every two preparations. Preparation of all the teeth specimens were performed under continuous water irrigation using rotary diamond cutting instruments of same shape, diameter, taper, tip configuration and grit sizes. The teeth specimens were randomly divided into one control group and one study group comprising 20 samples each (Group I and Group II, respectively). An axial groove of uniform size and shape was made on the prepared teeth in the study group (Group II) using a round end diamond bur (No. 111-014, Sharpcut, Dentsply Asia). The control group (Group I) was prepared without an axial groove.

#### Determination of axial surface area

**Figure 1:**
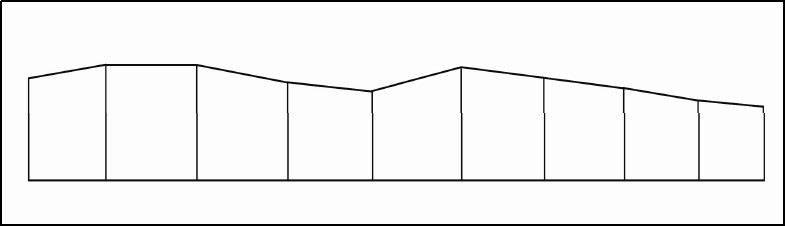
Polyline surface area.

The axial surface areas of the prepared teeth specimen were measured using a digital height measuring gauge, a digital Vernier caliper, and AutoCAD software. With the help of height measuring gauge (Mitutoyo No.192-609, Japan), occlusocervical height of each prepared tooth was measured clockwise at 10 different points around the prepared tooth. The circumference of the prepared tooth specimen was measured using an inextensible wire, which was wrapped around (approximately at the middle of the occlusocervical height) the prepared tooth specimen, and the required length was marked with a permanent marker. This length was measured using a Digital Vernier caliper with an accuracy of ± 0.02 mm (Absolute Digimatic, Mitutoyo, Japan). The recorded data of occlusocervical height at various locations and the circumference of the preparations were fed into an AutoCAD software program (AutoCAD, Autodesk, USA). The software measured the surface area as follows: the circumferential length of the prepared tooth specimen was plotted into 10 divisions. At each division, the measured height data was entered. The ten points of the height were joined to obtain a closed Polyline (Figure 1). The areas of the closed polyline were calculated by AutoCAD software.

#### Fabrication of crowns

Impression trays were fabricated for each prepared tooth. Impressions were made with Polyvinyl Siloxane (AquasiI TM, Dentsply Caulk, Milford, DE, USA) using the single mix technique. The impressions were poured in type IV die stone (Silky Rock Yellow, Whipmix, Louisville, KY, USA) to prepare working dies. The dies were painted with three coats of die-spacer (Pico-Fit, Renfert GmbH, Hilzingen, Germany) on the surface excluding the margin. Wax patterns for crowns were prepared with casting wax (Crown wax, BEGO, Germany), with a thickness of approximately 0.5 to 1 mm after lubrication of the dies with die lubricant (Dielube wax sep, Dentecon lnc., USA). A round sprue wax (Rewax, Renfurt GMbH, Hilzingen, Germany) shaped as a loop was attached to the center of the occlusal surface of each wax pattern parallel to the long axis of the prepared tooth to facilitate connection of the crown to a Universal testing machine. Wax patterns were sprued and then, invested and cast in non-precious casting alloy (Wirolloy, BEGO, Germany) with an electronic induction casting machine (Degutron, Degussa, AG, Germany). Castings were carefully divested and checked for seating discrepancies with a disclosing medium (Fit checker, GC Corporation, Tokyo, Japan.).

#### Luting of the crowns

The Groups I and II were divided into two subgroups of 10 samples each, IA, IB and IIA, IIB, respectively.

The subgroups IA and IIA were cemented with self-adhesive universal resin luting cement (RelyX Unicem Applicap, 3M ESPE, Germany). The specimens were air dried, taking care to avoid desiccation. The capsules were placed in a mixing unit (Promix, Dentsply) for 15 seconds. Using the Applicap applier, the crown was filled with cement and seated over the tooth with firm axial movement under digital pressure. Excess cement from the margin was removed with an explorer.

The subgroups IB and IIB were cemented with conventional multi-step dual cure luting cement (Calibra, Dentsply Caulk, Milford, DE, USA). Teeth were etched and two drops, each of Prime® and Bond NT® dual-polymerizing adhesive and auto-polymerizing activator were mixed, applied to the tooth surface for 20 seconds and air dried for 5 seconds, and then light polymerized for 10 seconds. A single coat of the same mix was applied to the internal surface of the crown and air dried for 5 seconds. Equal amounts of base and regular viscosity catalyst were mixed until uniform, and then a thin layer was applied to the intaglio surface of the crown. The crown was seated with a gentle, minimum finger pressure to reproduce clinical cementation. Finger pressure was slowly increased to ensure complete seating and each crown was maintained under pressure for 15 seconds. Thereafter, the assembled teeth and castings were placed in a loading device (Spring Dial™, Wagner Instruments, Greenwich, CT, USA) and subjected to an axial force of 20 kg per casting for 10 minutes. After removal of excess cement, all marginal areas were light polymerized for 20 seconds.

**Figure 2:**
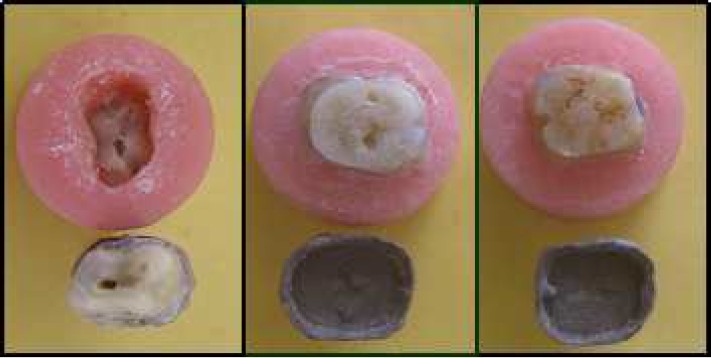
Failure modes of resin cement

The cemented specimens (Figure 2) were then stored in distilled water for 7 days at room temperature before subjecting them to a tensile load.

#### Testing for tensile bond stress

The testing of the specimens for tensile bond stress was performed with a Universal testing machine (Instron, model-4206, Canton Inc., USA). Each specimen was aligned in the center of attachment of the testing unit and special care was taken to ensure that each tooth was fixed with its long axis congruent with the loading axis of the testing apparatus to ensure that the tensile load was directed along the long axis of each specimen using a special jig designed for the purpose. A uniform tensile load was applied at a constant crosshead speed of 1 mm/min. The maximal tensile force used to separate the crown was recorded. In addition, the separated crowns and axial walls of the preparations were examined to observe the mode of failure. Four failure modes (Figure 2) were defined: 1) Root fractures were defined as cohesive failure of dentin while the crown remained luted in place. 2) Failure at the metal-cement interface was consid-ered when greater than 75% of the luting agent remained on the axial walls of the prepared tooth. 3) Failure at the dentin-cement interface was indicated when greater than 75% of cement was present on the internal metal surface of the casting. 4) When neither the dentin nor the casting had a clear preponderance of retained cement, i.e., less than 75% of cement was retained, the failure was defined as mixed.

The tensile bond stress of each specimen was calculated, dividing the recorded maximal tensile force by the total axial surface area of the prepared tooth.

$$\mathrm{Tensile}\;\mathrm{stress}\;\left(\sigma \right)\;=\;\frac{Maximum\;tensile\;force\;\left(F\right)}{Total\;surface\;area\;of\;Prepared\;tooth}$$

The results obtained from this study were statistically analyzed by “Mann-Whitney U” test. P value less than 0.05 was considered statistically significant and less than 0.01 was considered highly significant.

## Results

The results are tabulated in tables 1 and 2. Mann–Whitney U test showed a significant difference between the two resin cements in control and test groups (Z=2.72100 & 2.71900, respectively; P = 0.005 & 0.006, respectively) and no significant differences between the subgroups IA & IIA (Z = 1.15100, P = 0.88) and IB and IIB (Z = 1.26100, P = 0.18).

**Table 1:** Mean (SD) of tensile force (N) and strength (MPa) of tested groups.

Group	Tensile Force (N)	Tensile Strength(MPa)
IA	566.35(101.000)	5.76(0.392)
IIA	653.87(74.675)	5.93(0.751)
IB	518.69(50.405)	4.92(0.641)
IIB	618.1(90.202)	5.15(0.478)

No significant differences between groups of IA & IIA (Z = 1.15100, P = 0.88) and IB and IIB (Z = 1.26100, P = 0.18) were found. Significant difference between the two resin cements in control and test groups were revealed (Z = 2.72100 & 2.71900, respectively; P = 0.005 & 0.006, respectively).

**Table 2:** Modes of failure in the study groups

Cement	Group	Root fracture	Failure at Metal-Cement interface	Failure at Dentin-Cement interface	Mixed Failure
RelyX -	IA	6	0	4	0
Unicem	IIA	4	0	6	0
Calibra	IB	4	0	4	2
	I IB	2	0	4	4

The distribution of modes of failure in specimens luted with Calibra (Groups A) and RelyX Unicem (Groups B) resin cements are shown in table 2. Forty percent of the specimens luted with Calibra resin cement revealed failures in the dentin-cement interface, 20% failed by root fracture and 20% showed a mixed pattern of failure. Failed specimens cemented with RelyX Unicem showed 50% of failures by root fractures and 50% of fail-ures at the Dentin-Cement interface.

## Discussion

The results of this in-vitro study revealed marginal gains in the retentive strength of complete cast metal crowns in the study group (Group II) compared to the control group (Group I) (Table 1). However, this increase in retention was statistically insignificant. The results obtained in the present study corroborates with the findings of Potts et al.^6^ A possible explanation for the insignificant gain in retention associated with the addition of grooves may rest in the fact that placement of groove adds very little to the total surface area of the preparation.^6^ However, the tensile force and tensile stress values obtained for resin cements in this in-vitro study were significantly different than those obtained in the previous studies mentioned in the dental literature.^24^,^25^ The reasons for this disparity could be the use of different methods of standardization of specimens, and the use of dif-ferent resin luting cements.

Among the two resin cements used in this study, RelyX Unicem (3M ESPE) showed better retentive properties compared to that of Calibra (Dentsply). This increase in retentive strength was highly significant (Table 1) for both the control groups and the study groups. This may be attributed to the increased hydrophobicity and moisture tolerance by the RelyX Unicem self-adhesive resin cement. RelyX™ Unicem Self-Adhesive Universal Resin Cement’s neutral and hydrophobic end state helps to contribute to the cement’s integrity and durable high bond strength (RelyX Unicem Technical Product Profile, p.3-56). However, a clinical study may validate the long-term clinical outcome of the cement. The distribution modes of failure in specimens luted with Calibra resin cement revealed that most of the failures occurred at the dentin-cement interface. This observation was similar to those obtained by Uy et al^26^ and contrary to the study conducted by Browning WD et al.^13^ RelyX Unicem failed predominantly by root fractures and failure at the Dentin-Cement interface. This was contrary to the observations of Uy et al^26^ where the majority of failures occurred at the Crown-Cement interface. This pattern of failure probably explains the increased retentive strengths found with these luting agents. The excellent bonding of adhesive resin luting cements to base metal alloys has been reported by Ergin^17^ and Browning.^13^ The patterns of failure observed in the present study probably substantiate their findings. However, a more detailed observation of the pattern of failure under a SEM (Scanning Electron Microscope) would be necessary to draw definite conclusions. Many factors that influence the retention of complete cast metal crowns with varied magnitude are very difficult to control and standardize in an in-vitro study. However, all possible efforts were made to standardize the specimens used in the study. Intact molar teeth of approximately same size were vertically aligned using a Pindex System, so that their long axis passes through the area of furcation and the axial reduction was carried out on a milling machine using rotary diamond bur in order to standardize the degree of taper. In addition, a new rotary diamond bur was used for preparation of each specimen to standardize the surface texture. The ‘AutoCAD’ software was used to calculate the axial surface area to avoid manual imperfections. The force was converted to stress using the axial surface area of each preparation. The use of stress, rather than force, standardizes values so that they can be compared with results of future studies. Cementations of castings were performed randomly by a single operator to avoid bias.

The limitations of the current study include the two dimensional depiction of a three-dimensional object by the 2D AutoCAD software and naked eye observation of the pattern of failure in the cemented specimens. In addition, the unavoidable introduction of stress other than that of tensile nature during the testing procedure complicates the standardization.

For further studies, it is suggested to use a laser scanner and 3D AutoCAD software to measure the surface area of prepared teeth specimen and a SEM study for the mode of cement failure. Also, a clinical study may validate the long-term clinical success of the resin cements.

## Conclusion

Within the limitations of the study, the following conclusions can be drawn:

1. Axial groove has no statistically significant effect on the retention of complete cast metal crowns.
2. RelyX Unicem (3M ESPE) has better retentive strength when compared with Calibra (Dentsply Caulk).
3. The group of specimens cemented with RelyX Unicem failed predominantly by root fractures and failure at the dentin-cement interface while specimens cemented with Calibra exhibited most of the failures at dentin-cement interface.
